# Rutin and Gallic Acid Regulates Mitochondrial Functions via the SIRT1 Pathway in C2C12 Myotubes

**DOI:** 10.3390/antiox10020286

**Published:** 2021-02-13

**Authors:** Wei-Tang Chang, Shih-Chien Huang, Hsin-Lin Cheng, Shiuan-Chih Chen, Chin-Lin Hsu

**Affiliations:** 1 Department of Nutrition and Health Nutrition, Chinese Culture University, Taipei 11114, Taiwan; zwt6@ulive.pccu.edu.tw; 2 Department of Nutrition, Chung Shan Medical University, Taichung 40201, Taiwan; schuang@csmu.edu.tw (S.-C.H.); iamsamlee@livemail.tw (H.-L.C.); 3 Institute of Medicine and School of Medicine, Chung Shan Medical University, Taichung 40201, Taiwan; sccy399@gmail.com; 4 Department of Family and Community Medicine, Chung Shan Medical University Hospital, Taichung 40201, Taiwan; 5 Department of Nutrition, Chung Shan Medical University Hospital, Taichung 40201, Taiwan

**Keywords:** C2C12 myotubes, rutin, gallic acid, Sirtuin-1, mitochondria

## Abstract

Mitochondria are highly dynamic organelles, balancing synthesis and degradation in response to increases in mitochondrial turnover (i.e., biogenesis, fusion, fission, and mitophagy) and function. The aim of this study was to investigate the role of polyphenols in the regulation of mitochondrial functions and dynamics in C2C12 myotubes and their molecular mechanisms. Our results indicate that gallic acid and rutin are the most potential polyphenol compounds in response to 15 phenolic acids and 5 flavonoids. Gallic acid and rutin were associated with a significantly greater mitochondrial DNA (*cytochrome b* and *COX-II*), mitochondrial enzymatic activities (including citrate synthase and cytochrome *c* oxidase), and intracellular ATP levels in C2C12 myotubes. Moreover, gallic acid and rutin significantly increased the gene expressions of mitochondrial turnover in C2C12 myotubes. Our findings indicated that gallic acid and rutin may have a beneficial effect on mitochondrial dynamics via regulation of the SIRT1-associated pathway in C2C12 myotubes.

## 1. Introduction

The number of people affected by fatigue has continued to increase throughout the 21st century. Fatigue can manifest as feelings of tiredness, reduced energy levels, muscle weakness, and adverse effects on social relationships [1]. Skeletal muscle is an important organ involved in supporting the body, physical activity, and metabolic homeostasis [2,3,4]. Previous studies have defined skeletal muscle fatigue as a decline in the ability of muscle to generate force [5]. Mitochondria in the skeletal muscles produce nearly 90% of the cellular ATP required to maintain muscle activity through aerobic respiration and oxidative phosphorylation (OXPHOS) [6]. Therefore, mitochondrial function is a potential biomarker for the oxidative capacity and functionality muscle. Moreover, the maintenance of mitochondrial function is reportedly essential for maintaining the homeostasis of energy and metabolism [7,8,9].

Mitochondrial biogenesis is a complex process [10]. Previous studies have indicated that mitochondria are dynamic organelles involved in essential cellular functions. These functions require coordinated changes in the expression of numerous metabolic genes associated with mitochondrial biogenesis, fusion, fission, and mitophagy [11,12]. However, the mechanism of mitochondrial biogenesis remains largely unknown. Recently, silent information regulator two homolog 1 (SIRT1) has been implicated as a major regulator of mitochondrial biogenesis that directly interacts with the expression of peroxisome proliferator-activated receptor gamma coactivator-1 alpha (PGC-1α) in the skeletal muscles [13]. SIRT1 and PGC-1α are key mitochondrial transcription factors responsible for the regulation of mitochondrial DNA and mitochondrial dynamics-related genes (i.e., *NRF1*, *TFAM*, *OXPHOS*, *Mfn1*, and *LC3II*) [14,15,16]. As mentioned above, activated SIRT1 and PGC-1α pathways are validated biomarkers for mitochondrial functions in skeletal muscle, which can upregulate both an individual’s aerobic capacity and exercise performance [17].

Phenolic acids and flavonoids constitute the common polyphenols, which are ubiquitously present in plants. These compounds reportedly have important biological and pharmacological properties and may benefit human health. Polyphenols have been widely studied for their antioxidant, anti-inflammatory, anti-carcinogenic, and anti-obesity properties [18,19,20,21]. In addition, they can provide protection against oxidative stress and related diseases [20,22,23]. Moreover, polyphenols have been reported to be potential activators of SIRT1 or PGC-1α and are used as therapies for numerous biological activities; however, few studies have investigated their effects on exercise performance or mitochondrial biogenesis in vitro and animal models [24,25,26]. Therefore, we aimed to investigate the in vitro effects of polyphenols on the regulation of mitochondrial biogenesis and functionality in C2C12 myotubes.

## 2. Materials and Methods

### 2.1. Materials

The analytic phenolic components as followed: resveratrol, hydroxybenzoic acids (i.e., salicylic acid, *p*-hydroxybenzoic, syringic acid, gallic acid, vanillic acid, 3,4-dimethoxybenzoic acid, protocatechuic acid, and gentisic acid), hydroxycinnamic acids (i.e., *p*-coumaric acid, *o*-coumaric acid, *m*-coumaric acid, caffeic acid, ferulic acid, sinapinic acid, and chlorogenic acid), and flavonoids (i.e., naringenin, hesperidin, naringin, kaempferol, and rutin) were purchased from Sigma-Aldrich (Saint Louis, MO, USA). The compounds were dissolved in dimethyl sulfoxide (DMSO) and stored at −20 °C. Reagent chemicals used in the present study were of analytical grade. 

### 2.2. C2C12 Myoblast Cell Culture

The mouse C2C12 myoblast cells (BCRC No: 60083) obtained from the Food Industry Research and Development Institute (Hsinchu, Taiwan), were maintained in Dulbecco’s Modified Eagle Medium (DMEM) contained 10% fetal bovine serum (FBS), sodium bicarbonate (1.5 g/L), penicillin (100 units/mL), and streptomycin (100 μg/mL) in a humidified 37 °C incubator with atmosphere of 5% CO_2_. C2C12 myoblast cells were transferred into the well of a 12-well culture plates and 10 cm petri dishes. After reaching confluence, C2C12 myoblast cells were then differentiated into myotubes over eight days, and then the medium was changed in DMEM contained 2% horse serum, sodium bicarbonate (1.5 g/L), penicillin (100 units/mL), and streptomycin (100 μg/mL) for differentiation. The culture medium was refreshed every 2-days during differentiation.

### 2.3. Creatine Kinase Activity

Creatine kinase (CK) is induced to high levels during muscle cell differentiation [27,28]. CK activity was assessed in the C2C12 myotube cells lysates with a CK activity assay kit (Biovision, Mountain View, CA, USA). The protocol was performed as specified by the manufacturer. CK activity was detected spectrophotometrically at 450 nm with a microplate spectrophotometer (Molecular Devices, Sunnyvale, CA, USA) every 5 min for 20 min. 

### 2.4. Cell Cytotoxicity Test

Cytotoxicity was assessed through the outflow of cytosolic enzyme, lactate dehydrogenase (LDH) presents in culture medium was detected with enzymatic activity following cellular damage [29]. The C2C12 myotubes were incubated with resveratrol, the hydroxybenzoic acids, hydroxycinnamic acids, and flavonoids (50 and 100 μM) for 48 h. Triton X-100 (0.1% in medium) was used to create a cytotoxic model in C2C12 cells, and served as positive control. Cellular toxicity was determined by the level of LDH release into the medium and was analyzed according to the manufacturer’s protocol of LDH cytotoxicity assay kit (Biovision, Mountain View, CA, USA). The amount of LDH released (%) was detected spectrophotometrically at 450 nm with a microplate spectrophotometer (Molecular Devices, Sunnyvale, CA, USA) for 30 min.

### 2.5. mtDNA Quantification

C2C12 myotubes were treated with resveratrol, the hydroxybenzoic acids, hydroxycinnamic acids, and flavonoids (50 and 100 μM) for 24 h. Genomic DNA was extracted as procedure described in AccuPrep^®^ genomic DNA extraction kit (Bioneer, Daejeon, Korea). DNA concentration and quality were checked spectrophotometrically (260/280 nm and 260/280 ratio) with nanophotometer spectrophotometer (IMPLEN, Schatzbogen, München, Germany). The specific primer for amplification of analytic genes were as follow forward (F) and reverse (R) primer sequence described: β-actin-F: 5′-TTGTAACCAACTGGGACGATATGG-3′, β-actin-R: 5′-CTAATAGACGAAGTTCACCTGG-3′ (Accession numbers: X03672); cytochrome b-F: 5′-TTCGCAGTCATAGCCACAG-3′, cytochrome b-R: 5′-AGATGAAGTGGAAAGCGAAG-3′ (Accession numbers: AB033699); cytochrome c oxidase subunit II, (COX-II)-F: 5′-CAAGACGCTACATCACCTATC-3′, cytochrome c oxidase subunit II, (COX-II)-R: 5′-CTAATAGACGAAGTTCACCTGG-3′ (Accession numbers: X68508). To quantify the relative changes in target genes expression, amplifications were carried out using a StepOne^TM^ real-time RT-PCR system (Applied Biosystems, Carlsbad, CA, USA). PCR reaction mixture consists of 10 μL SYBR Green Master Mixes, 1 μL specific primer pairs (300 nM), 1 μL DNA template, and sterilised deionized water to reach a total volume of 20 μL. Amplification were processed using manufacturer’s instructions: initiated at 95 °C for 10 min, followed by 40 cycles of 95 °C for 15 s, 60 °C for 60 s, and 25 °C forever in brief. The β-actin served as a loading control for normalization of relative gene expression levels.

### 2.6. Mitochondrial Staining and Quantification

To determine the mitochondrial content in the C2C12 myotubes, a 10-N-nonyl acridine orange (NAO) staining method (Sigma, St. Louis, MO, USA) was conducted. C2C12 myotubes were incubated with resveratrol, rutin, gallic acid, *o*-coumaric acid, and syringic acid (50 μM) for 24 h. After incubating with each treatment, the C2C12 myotubes were exposed to 500 nM NAO dyes at 37 °C for 2 h. Followed by washing the cells with phosphate buffered saline (PBS, PH 7.4), and fixed for 20 min in 10% neutral-buffered formalin at room temperature. Subsequently, we carefully removed the neutral-buffered formalin, the cells nuclei were counterstained with DAPI for 10 min and then washed extensively with PBS. The florescence signal from the mitochondrial and nuclear morphological feature were detected with fluorescence microscope (AE31, Richmond, Canada) at × 200 magnification. The cells were incubated with lysis buffer (1% Triton X-100 in PBS) on the shaker softly. After 10 min of lysation, cells received DMSO for another 10 min incubation. For quantitative analysis, the cells lysates were subjected to Flexstation 3 fluorescence plate reader with an excitation/emission wavelength at 488/550 nm.

### 2.7. Mitochondrial Enzyme Activity

To determine enzyme activity of mitochondria in C2C12 myotubes, the cells were treated with the indicated concentration (25 and 50 μM) of resveratrol, rutin, gallic acid, *o*-coumaric acid, and syringic acid for 24 h. Following the manufacturer’s instruction, the mitochondrial-enrich fractions were extracted by Sciencell™ mitochondria isolation kit (Sciencell, Carlsbad, CA, USA). The enzyme activity of citrate synthase and cytochrome c oxidase in the harvested mitochondrial fraction were determined by Sciencell™ citrate synthase assay kit (Cat NO. 8318) and Sciencell™ cytochrome c oxidase assay kit (Cat NO. 8278), respectively. Citrate synthase and cytochrome *c* oxidase activity were measured spectrophotometrically at 412 nm and 550 nm, respectively, with a VersaMax tunable microplate reader (Molecular Devices, Sunnyvale, CA, USA).

### 2.8. Intracellular ATP Content

To determine intracellular energy levels in C2C12 myotubes, the cells were treated with the indicated concentration (25 and 50 μM) of resveratrol, rutin, gallic acid, *o*-coumaric acid, and syringic acid for 24 h. Following the manufacturer’s instruction, the ATP content was luminometrically detected by Flexstation 3 tunable microplate reader (Molecular Devices, Sunnyvale, CA, USA) for 2 min.

### 2.9. RNA Isolation, cDNA Synthesis, and Real-Time RT-PCR

After the treatment of rutin (25 μM) for 1 h and 9 h and gallic acid (25 μM) for 3 h and 12 h, total RNA was extracted from C2C12 myotubes using TRIzol reagent (Life Technologies, Rockville, MD, USA), according to the manufacturer’s instructions. Total RNA (2 μg) was subjected to synthesized complementary DNA (cDNA) by a high-capacity RNA-to-cDNA reverse transcription kit (Applied Biosystems, Foster City, CA, USA), according to the manufacturer’s instructions. The specific primer for amplification of analytic genes were the forward (F) and reverse (R) primer sequence described: β-actin-F: 5′-TTGTAACCAACTGGGACGATATGG-3′, β-actin-R: 5′-CTAATAGACGAAGTTCACCTGG-3′ (Accession numbers: X03672); Silent information regulator two homolog 1 (SIRT1)-F: 5′-TGTGAAGTTACTGCAGGAGTGTAAA-3′, Silent information regulator two homolog 1 (SIRT1)-R: 5′-GCATAGATACCGTCTCTTGATCTGAA-3′ (Accession numbers: Q923E4); peroxisome proliferator-activated receptor gamma coactivator 1α (PGC-1α)-F: 5′-GTCAACAGCAAAAGCCACAA-3′, peroxisome proliferator-activated receptor gamma coactivator 1α (PGC-1α)-R: 5′-TCTGGGGTCAGAGGAAGAGA-3′ (Accession numbers: O70343); nuclear respiratory factor 1 (NRF1)-F: 5′-ACCCTCAGTCTCACGACTAT-3′, nuclear respiratory factor 1 (NRF1)-R: 5′-GAACACTCCTCAGACCCTTAAC-3′ (Accession numbers: BC005410); estrogen-related receptor α (ERRα)-F: 5′-ACTGCCACTGCAGGATGAG-3′, estrogen-related receptor α (ERRα)-R: 5′-CACAGCCTCAGCATCTTCAA-3′ (Accession numbers: NM_007953), (forward); mitochondrial transcription factor A (TFAM)-F: 5′-AAGGGAATGGGAAAGGTAGAG-3′, mitochondrial transcription factor A (TFAM)-R: 5′-ACAGGACATGGAAAGCAGATTA-3′(Accession numbers: NM_009360); NADH dehydrogenase (ubiquinone) 1 beta subcomplex 3 (NDUFB 3)-F: 5′-AAGGGACGCCATTAGAAACG-3′, NADH dehydrogenase (ubiquinone) 1 beta subcomplex 3 (NDUFB 3)-R: 5′-TACCACAAACGCAGCAAACC-3′ (Accession numbers: NM_025348); succinate dehydrogenase complex subunit B (SDHB)-F: 5′-TGGTGGAACGGAGACAAGTA-3′, succinate dehydrogenase complex subunit B (SDHB)-R: 5′-TGGCAGCGGTAGACAGAGAA-3′ (Accession numbers: Q9CQA3); cytochrome *c* oxidase subunit 5b (COX5b)-F: 5′-CGTCCATCAGCAACAAGAGA-3′, cytochrome *c* oxidase subunit 5b (COX5b)-R: 5′-AGATAACACAGGGGCTCAGT-3′ (Accession numbers: AAH96048); ubiquinol-cytochrome *c* reductase complex I (UQCRC1)-F: 5′-GGGGCAAAAACATCCTTAGG-3′, ubiquinol-cytochrome *c* reductase complex I (UQCRC1)-R: 5′-ATCCGGCTCTCCCACTCAGC-3′ (Accession numbers: NM_025407); ATP synthase, H+ transporting, mitochondrial Fo complex, subunit E (ATP51)-F: 5′-CCCCTGCTGAAATCCCTACA-3′, ATP synthase, H^+^ transporting, mitochondrial Fo complex, subunit E (ATP51)-R: 5′-TAAAACCACATCCACACCTC-3′ (Accession numbers: AAH31384); myosin heavy chain I (MyHC I)-F: 5′-GTCCAAGTTCCGCAAGGT-3′, myosin heavy chain I (MyHC I)-R: 5′-CCACCTAAAGGGCTGTTG-3′ (Accession numbers: NM_080728); myosin heavy chain IIa (MyHC IIa)-F: 5′-CGATGATCTTGCCAGTAATG-3′, myosin heavy chain IIa (MyHC IIa)-R: 5′-ATAACTGAGATACCAGCG-3′ (Accession numbers: NM_144961); myosin heavy chain IIb (MyHC IIb)-F: 5′-CAATCAGGAACCTTCGGAACAC-3′, myosin heavy chain IIb (MyHC IIb)-R: 5′-GTCCTGGCCTCTGAGAGCAT-3′ (Accession numbers: XM_126119); mitofusin 1 (Mfn1)-F: 5′-GCTGTCAGAGCCCATCTTTC-3′, mitofusin 1 (Mfn1)-R: 5′-CAGCCCACTGTTTTCCAAAT-3′ (Accession numbers: NM_024200); mitofusin 2 (Mfn2)-F: 5′-GCCAGCTTCCTTGAAGACAC-3′, mitofusin 2 (Mfn2)-R: 5′-GCAGAACTTTGTCCCAGAGC-3′ (Accession numbers: NM_001355590); dynamin-related protein 1 (DRP1)-F: 5′-GTTCCACGCCAACAGAATAC-3′, dynamin-related protein 1 (DRP1)-R: 5′-CCTAACCCCCTGAATGAAGT-3′ (Accession numbers: AB079133); mitochondrial fission protein 1 (Fis1)-F: 5′-AAGTATGTGCGAGGGCTGT-3′, mitochondrial fission protein 1 (Fis1)-R: 5′-TGCCTACCAGTCCATCTTTC-3′ (Accession numbers: NM_025562); optic atrophy 1 (Opa1)-F: 5′-CAGCTGGCAGAAGATCTCAAG -3′, optic atrophy 1 (Opa1)-R: 5′-TATGAGCAGGATTTTGACACA -3′ (Accession numbers: BC138665); Bcl-2 nineteen-kilodalton interacting protein 3 (Bnip3)-F: 5′-GCTCCTGGGTAGAACTGCAC-3′, Bcl-2 nineteen-kilodalton interacting protein 3 (Bnip3)-R: 5′-GCTGGGCATCCAACAGTATT-3′ (Accession numbers: FM995532); Beclin1-F: 5′-CCGGGCGATGGGAACTCTGGA-3′, Beclin1-R: 5′-CCTCCATGCCTCAGGAGCCCG-3′ (Accession numbers: NM_172669); autophagy-related protein 5 (Atg5)-F: 5′-AGCAGCTCTGGATGGGACTGC-3′, autophagy-related protein 5 (Atg5)-R: 5′-GCCGCTCCGTCGTGGTCTGA-3′ (Accession numbers: NM_053069); autophagy-related protein 7 (Atg7)-F: 5′-CCTGCACAACACCAACACAC-3′, autophagy-related protein 7 (Atg7)-R: 5′-CACCTGACTTTATGGCTTCCC-3′ (Accession numbers: NP001240646); microtubule-associated protein light chain 3II (LC3II)-F: 5′-CACTGCTCTGTCTTGTGTAGGTTG-3′, microtubule-associated protein light chain 3II (LC3II)-R: 5′-TCGTTGTGCCTTTATTAGTGCATC-3′ (Accession numbers: NM_026160); mitochondrial ubiquitin ligase 1 (Mul1)-F: 5′-AGGGCATTCTTTCAGAAGCA-3′, mitochondrial ubiquitin ligase 1 (Mul1)-R: 5′-GGGGTGGAACTTCTCGTACA-3′ (Accession numbers: NM_026689). To quantify the relative changes in target gene expression, amplifications were carried out using a StepOne^TM^ real-time RT-PCR system (Applied Biosystems, Carlsbad, CA, USA). PCR reaction mixture consisted of 10 μL SYBR Green Master Mixes, 1 μL specific primer pairs (300 nM), 1 μL DNA template, and sterilised deionized water to reach a total volume of 20 μL. Amplification were processed using manufacturer’s instructions: initiated at 95 °C for 10 min, followed by 40 cycles of 95 °C for 15 s, 60 °C for 60 s, and 25 °C forever in brief. The β-actin served as a loading control for the normalization of relative gene expression levels. 

### 2.10. SIRT1 Knockdown

For silencing SIRT1 protein expression, SIRT1 siRNA was purchased from LifeTechnologies (Tokyo, Japan). The C2C12 myotubes were plated in 6-well culture plates overnight. Following the manufacturer’s protocol: a total of 30 pmol SIRT1 siRNA were transfected into C2C12 myotubes using lipofectamine RNAiMAX transfection reagent (Invitrogen, CA, USA), and incubated in serum-free medium (Opti-MEM) for 48 h. Subsequently, the C2C12 myotubes were treated with 25 μM rutin for an additional 1 h and 9 h and gallic acid for an additional 3 h and 12 h. The total RNA sample preparation and followed real-time PCR amplification procedure were detailed as mentioned above.

### 2.11. Statistical Analysis

In the present study, data were expressed as the mean ± SD of three independent experiments. For all of the measurements, statistical difference was ascertained using one-way ANOVA with post hoc Duncan’s test. *p* value under 0.05 was considered to indicate statistically significant.

## 3. Results

### 3.1. Measurement of Creatine Kinase (CK) Activity in C2C12 Myotubes

**Figure 1:**
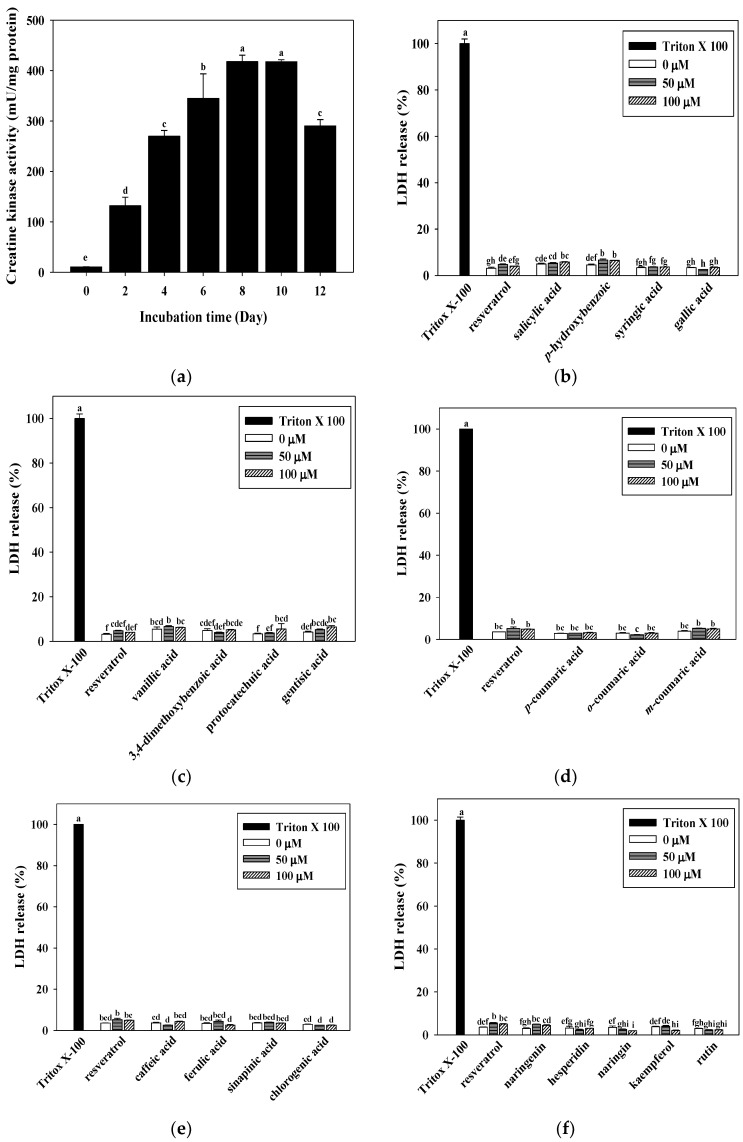
Effects of differentiation time course on creatine kinase (CK) activity (**a**) and hydroxybenzoic acids, hydroxycinnamic acids, and flavonoids on cytotoxicity (**b**–**f**) in C2C12 myotubes. C2C12 myotubes were differentiated with 0–12 days at 37 °C in a humidified 5% CO_2_ incubator. Cytotoxicity effect on C2C12 myotubes were incubated with 0–100 μM of resveratrol, hydroxybenzoic acids, hydroxycinnamic acids, and flavonoids for 48 h. Lactate dehydrogenase (LDH) activity (%) is expressed as 0.1% Triton X-100 at 100%. The reported values are the means ± SD (*n* = 3). Mean values with different letters were significantly different (*p* < 0.05).

The effects on C2C12 differentiation were analyzed by the measurement of creatine kinase (CK) activity. CK activity is a well-described marker of C2C12 myotube differentiation [30]. As shown in Figure 1a, the activity of CK was found to rise with the incubation time (*p* < 0.05), and a stronger increase was observed between days 8 and 10 (417.98 and 417.73 mU/mg of protein, respectively) (*p* < 0.05).

### 3.2. Determination of Cell Cytotoxicity from LDH Release for Resveratrol, Hydroxybenzoic Acids, Hydroxycinnamic Acids, and Flavonoid Cytotoxicity in C2C12 Myotubes

LDH released into the cell culture media was used as a marker of cell damage. As a cytotoxicity test, the C2C12 myotubes were incubated in the presence of 0, 50, or 100 μM of resveratrol, the hydroxybenzoic acids, hydroxycinnamic acids, and flavonoids for 48 h to determine the cellular damage. Cytotoxicity was assessed in the C2C12 myotubes by the level of LDH activity. As shown in Figure 1b–f, following the exposure of C2C12 myotubes to resveratrol, the hydroxybenzoic acids, hydroxycinnamic acids, and flavonoids, there was a slight increase (less than 20%) in LDH release (i.e., no significant cytotoxicity) compared to the C2C12 myotubes treated with Tritox-X 100. The concentrations of resveratrol, the hydroxybenzoic acids, hydroxycinnamic acids, and flavonoids (50 and 100 μM) used did not exhibit any cytotoxic effect on the C2C12 myotubes. Therefore, these concentrations were used for all follow-up analyses.

### 3.3. Effects of Resveratrol, the Hydroxybenzoic Acids, Hydroxycinnamic Acids, and Flavonoids on Mitochondrial DNA Gene Expressions in C2C12 Myotubes

**Figure 2:**
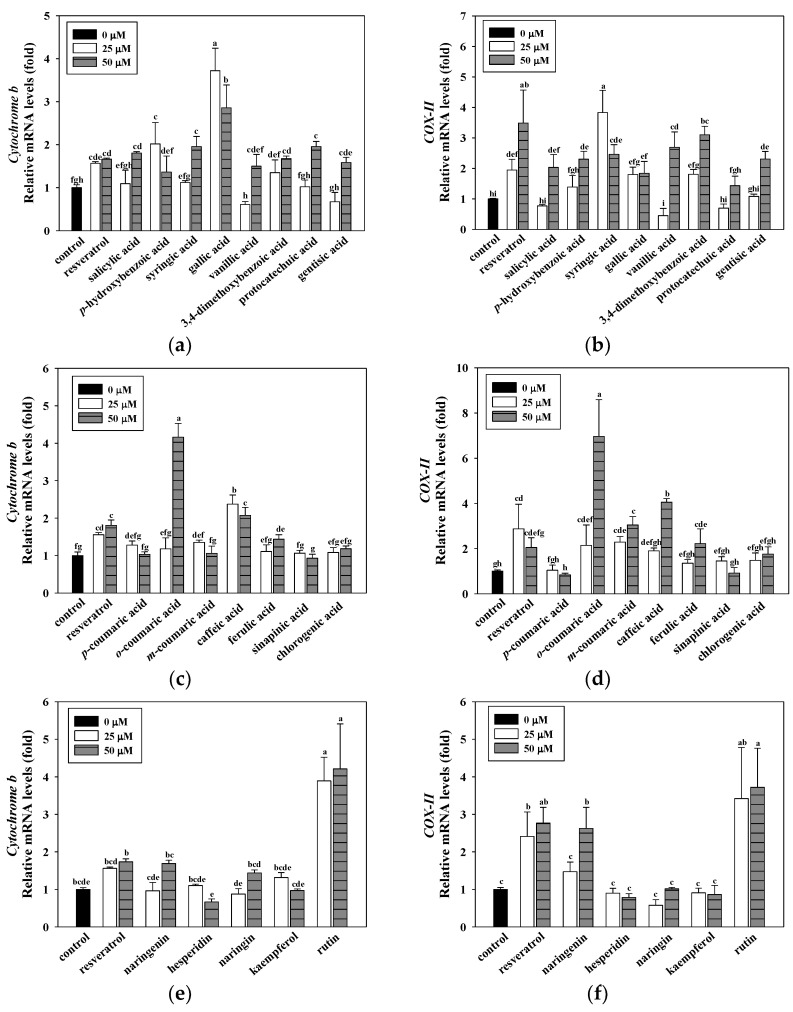
Effects of hydroxybenzoic acids (**a**,**b**), hydroxycinnamic acids (**c**,**d**), and flavonoids (**e**,**f**) on mitochondrial gene expressions in C2C12 myotubes. C2C12 myotubes were incubated with 25 and 50 μM of resveratrol and hydroxybenzoic acids for 24 h. Resveratrol was used as positive control. Control means C2C12 myotubes at 0.1% DMSO was used as a solvent control. The reported values are the means ± SD (*n* = 3). Mean values with different letters were significantly different (*p* < 0.05).

Resveratrol, a well-known potent activator of mitochondrial function, is used as a positive control in studies of various diseases [8]. Previous studies have showed that resveratrol significantly increased the expression of mitochondrial DNA genes and the activities of mitochondrial enzymes in C2C12 myotubes. Therefore, in the follow-up experiments of this study, resveratrol will be used as a positive control [31].The screening was conducted using the mitochondrial DNA content regarding the relative gene expression of *cytochrome b* and *cytochrome c oxidase subunit II* (*COX-II*) in the C2C12 myotubes. Figure 2. shows the effects of resveratrol, the hydroxybenzoic acids, hydroxycinnamic acids, and flavonoids on mitochondrial DNA gene expressions in the C2C12 myotubes. The results demonstrated that some phenolic acids and flavonoids significantly increased the expressions of *cytochrome b* and *COX-II* in the C2C12 myotubes. The data revealed that gallic acid, *o*-coumaric acid, and rutin induced the highest up-regulation of *cytochrome b* (3.72, 4.16, and 4.21-fold, respectively). Moreover, syringic acid, *o*-coumaric acid, and rutin significantly increased gene expressions of *COX-II* (3.83, 6.96, and 3.72-fold, respectively) among the 15 phenolic acids and five flavonoids that were tested. According to the above experimental results, resveratrol, gallic acid, syringic acid, *o*-coumaric acid, and rutin can significantly increase mitochondrial DNA content when compared to other compounds. Therefore, these compounds will be used in subsequent experiments.

### 3.4. Effects of Resveratrol, Syringic Acid, Gallic Acid, o-Coumaric Acid, and Rutin on Mitochondrial Content, Enzyme Activity, and Intracellular ATP Content in the C2C12 Myotubes

**Figure 3:**
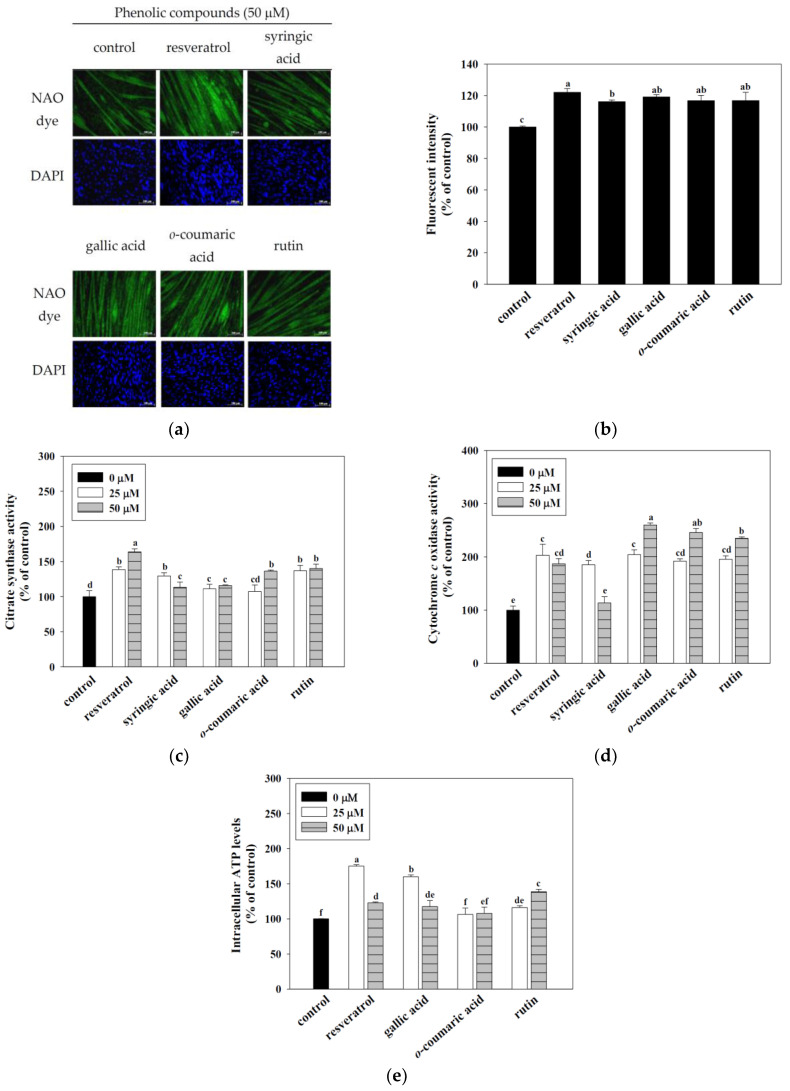
Effects of resveratrol, syringic acid, gallic acid, *o*-coumaric acid, and rutin on mitochondrial content (**a**,**b**) mitochondrial enzyme activities (**c**,**d**), and intracellular ATP levels (**e**) in C2C12 myotubes. C2C12 myotubes were incubated with 50 μM resveratrol, syringic acid, gallic acid, *o*-coumaric acid, and rutin for 24 h at 37 °C in a humidified 5% CO_2_ incubator. Representative NAO dye and DAPI staining of the C2C12 myotubes are shown at 200× magnification. For intracellular ATP levels, C2C12 myotubes were incubated with 25 and 50 μM of resveratrol, gallic acid, *o*-coumaric acid, and rutin for 24 h at 37 °C in 5% CO_2_ incubator. ATP concentrations were normalized to control. The enzyme activities of citrate synthase and cytochrome c oxidase are expressed as a percentage, with the value of control set at 100%. The reported values are the means ± SD (*n* = 3). Mean values with different letters were significantly different (*p* < 0.05).

The mitochondrial abundance in the C2C12 myotubes was measured using 10-N-nonyl acridine orange (NAO) dye. The results demonstrated that resveratrol, syringic acid, gallic acid, *o*-coumaric acid, and rutin significantly increased the mitochondrial content in C2C12 myotubes compared to the control (*p* < 0.05) (Figure 3a,b). Mitochondrial enzyme (i.e., citrate synthase and cytochrome *c* oxidase) activities appear to have an important role in ATP production. Therefore, phytochemicals significantly increased the mitochondrial enzymes activity, acting as an indicator of mitochondrial function. According to the citrate synthase activity results, resveratrol, syringic acid, gallic acid, rutin (25 and 50 μM) and *o*-coumaric acid (50 μM), compared to control (1 unit/mg mitochondria), can significantly increase the activity of citrate synthase (*p* < 0.05). Similarly, the results of cytochrome *c* oxidase activity show that resveratrol, gallic acid, *o*-coumaric acid, rutin (25 and 50 μM), and syringic acid (25 μM) compared to control (1 unit/mg mitochondria), can significantly increase the activity of cytochrome *c* oxidase (*p* < 0.05). According to the above results, resveratrol, gallic acid, *o*-coumaric acid, and rutin have a better effect on mitochondrial enzyme activities than syringic acid (*p* < 0.05) (Figure 3c,d). Therefore, resveratrol, gallic acid, *o*-coumaric acid, and rutin will be used for ATP content analysis. Mitochondria plays an important role in energy production. Therefore, effects of resveratrol, gallic acid, *o*-coumaric acid, and rutin on the total ATP content in the C2C12 myotubes. The results demonstrated that the C2C12 myotubes treated with 25 and 50 μM of resveratrol, gallic acid, and rutin significantly increased the total intracellular ATP content (*p* < 0.05) (Figure 3e). In addition, gallic acid and rutin have outstanding performance in mitochondrial DNA, mitochondrial number, enzyme activities, and ATP content. Therefore, gallic acid and rutin will be used for the follow-up molecular mechanism discussion in this study.

### 3.5. Gallic Acid and Rutin Induced Mitochondrial-Related Gene Expressions in C2C12 Myotubes

**Figure 4:**
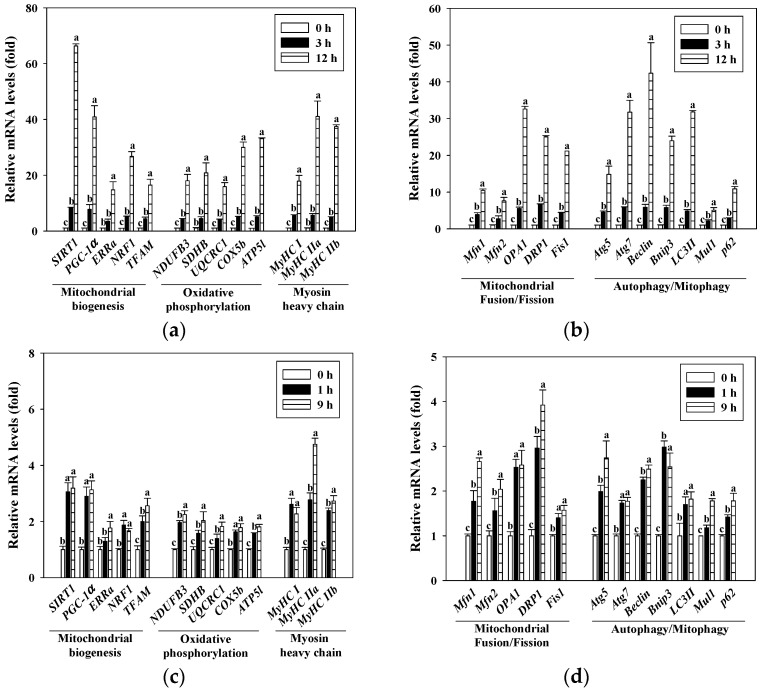
Effects of gallic acid (**a**,**b**) and rutin (**c**,**d**) on gene expressions of mitochondrial biogenesis, oxidative phosphorylation, myosin heavy chain, mitochondrial fusion/fission, and autophagy/mitophagy in C2C12 myotubes. C2C12 myotubes were incubated with 25 μM of gallic acid (3 and 12 h) or rutin (1 and 9 h) at 37 °C in a humidified 5% CO_2_ incubator. The reported values are the means ± SD (*n* = 3). Mean values with different letters were significantly different (*p* < 0.05).

Real-time RT-PCR was performed to determine the levels of mitochondrial-related gene expressions in the C2C12 myotubes. Figure 4a,b shows the effect of gallic acid on the gene expressions of mitochondrial biogenesis (i.e., *SIRT1*, *PCG-1α*, *ERRα*, *NRF1*, and *TFAM*), oxidative phosphorylation (i.e., *NDUFB3*, *SDHB*, *UQCRC1*, *COX5b*, and *ATP5l*), myosin heavy chain (i.e., *MyHC I*, *MyHC IIa*, and *MyHCIIb*), mitochondrial fusion/fission (i.e., *Mfn1*, *Mfn2*, *OPA1*, *DRP1*, and *Fis1*), and autophagy/mitophagy (i.e., *Atg5*, *Atg7*, *Beclin*, *Bnip3*, *LC3II*, *Mul1*, and *p62*) in the C2C12 myotubes. The gene expressions of mitochondrial biogenesis, oxidative phosphorylation, myosin heavy chain, mitochondrial fusion/fission, and autophagy/mitophagy were significantly increased when gallic acid (25 μM, 3 h and 12 h) was added to the C2C12 myotubes (*p* < 0.05). Figure 4c,d show that rutin (25 μM, 1 h and 9 h) significantly increased the gene expressions of mitochondrial biogenesis, oxidative phosphorylation, myosin heavy chain, mitochondrial fusion/fission, and autophagy/mitophagy in the C2C12 myotubes (*p* < 0.05).

### 3.6. Activition of Mitochondrial Functions by Gallic Acid and Rutin via the SIRT1 Associated Pathway

**Figure 5:**
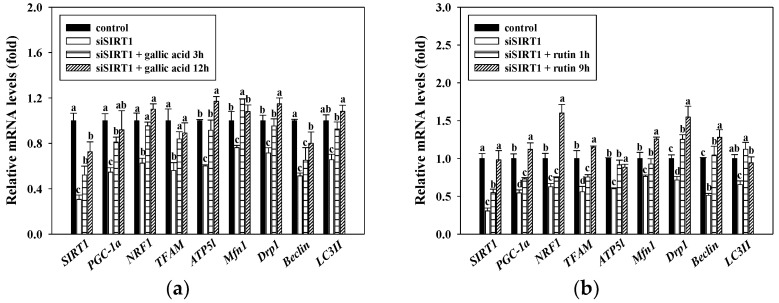
Effects of gallic acid (**a**) and rutin (**b**) on gene expressions of mitochondrial functions with SIRT1 siRNA transfection in C2C12 myotubes. C2C12 myotubes were transfected with SIRT1 siRNA by using Lipofectamine^®^ RNAiMAX reagent. After transfection, cells were treated with 25 μM of gallic acid (3 and 12 h) or rutin (1 and 9 h) at 37 °C in a humidified 5% CO_2_ incubator. The reported values are the means ± SD (*n* = 3). Mean values with different letters were significantly different (*p* < 0.05).

SIRT1 is reportedly implicated in energy production partically through mitochondrial biogenesis. To confirm whether SIRT1 mediates the effect of gallic acid and rutin on mitochondrial biogenesis, we silenced SIRT1 gene expression in C2C12 myotubes using an SIRT1-specific siRNA. The results demonstrated that the SIRT1 siRNA transfection successfully decreased *SIRT1* gene expression by approximately 30%. As expected, the mRNA expressions of *PGC-1α*, *NRF1*, *TFAM*, *ATP5l*, *Mfn1*, *DPR1*, *Beclin*, and *LC3II* was reduced by SIRT1-specific siRNA (*p* < 0.05). Furthermore, the protective effects of gallic acid and rutin on mitochondrial-related gene expressions were abolished by SIRT1-specific siRNA in the C2C12 myotubes (*p* < 0.05) (Figure 5a,b).

## 4. Discussion

Fatigue is commonly experienced and can cause tiredness in all aspects of the human body. In addition, fatigue has a negative impact on lifestyle, exercise endurance, work performance, and social relationships. Therefore, improvement in muscle strength and endurance are effective ways to improve the symptoms of muscle fatigue. The C2C12 cell line is commonly used as an in vitro model to study skeletal muscle performance, fibers, and contractibility [32,33]. CK activity is an established C2C12 differentiation marker, and is markedly elevated between days 8 and 10 of C2C12 myotube differentiation (Figure 1a). Mature C2C12 myotubes after eight days of differentiation are chosen for subsequent experimental analysis (i.e., mitochondrial content, enzyme activity, and intracellular ATP content). 

Mitochondria are the main power stations of cells, and are regulators of diverse cellular activities. In addition, mitochondria play an important role in cellular energy homeostasis. Due to the specialized function of mitochondria in the regulation of energy balance and metabolism, mitochondrial dysfunction is not surprisingly related to various diseases, including muscle weakness, metabolic disorders, cardiac disease, and aging. Therefore, maintaining the normal function of mitochondria plays an important role in sustaining human health. In our screening experiments, we used an assessment of mitochondrial DNA as an indicator of C2C12 myotube functionality. Natural products (NPs) are often defined as “molecules obtained from natural sources, which exhibit biological activities”. Phenolic acids (e.g., hydroxybenzoic acids and hydroxycinnamic acids) and flavonoids (e.g., isoflavonoids, flavanones, flavanols, flavonols, flavones, and anthocyanidins) are common forms of natural compounds. They are known to have important biological and pharmacological properties and may be beneficial to human health [21,23,34,35,36]. However, relatively few studies have reported that improvements in mitochondrial function are related to enhanced exercise performance [25,26]. Sirtuins are important histone deacetylases involved in NAD^+^-dependent deacetylation. Sirtuins activation is involved in a variety of metabolic-related diseases (such as type 2 diabetes, aging process, and inflammation); therefore, it is an important target category for recent research. It is known that NPs, including flavonoids, tanikolide, xanthone, resveratrol, bichalcones, and alkaloids are important sirtuins modulators [37]. Resveratrol, a SIRT1 activator, is widely known for its biological functions, including anti-oxidative, anti-cancer, and longevity properties [38,39,40]. It has also been reported that resveratrol acts as an exercise mimetic through increasing the activity of SIRT1 and AMPK [41]. Therefore, we used resveratrol as a positive control for mitochondrial functions. Our data revealed that the treatment of C2C12 myotubes with phenolic acids and flavonoids (50 and 100 μM) exhibited no cytotoxicity (<20% LDH release) (Figure 1b–f). Moreover, our results showed that resveratrol, gallic acid, syringic acid, *o*-coumaric acid, and rutin can induce optimal up-regulation of mitochondrial gene expressions (i.e., *cytochrome b* and *cytochrome c oxidase subunit II*) among the 15 phenolic acids and 5 flavonoids we tested (Figure 2). The observations mentioned above seem to have the same effect as that of resveratrol, as gallic acid, syringic acid, *o*-coumaric acid, and rutin increased the number of mitochondria in the C2C12 myotubes. We conducted follow-up mitochondrial enzyme activity experiments to help select an optimal candidate for enhancing mitochondrial functionality. 

Citrate synthase or cytochrome *c* oxidase are key mitochondrial enzymes that have been widely used as indices of mitochondrial oxidative capacity [42,43]. Assessing the activity level of mitochondrial enzymes is an approach used to investigate mitochondrial function in skeletal muscle. Our data indicate that the treatment of C2C12 myotubes with gallic acid and rutin significantly increased the mitochondrial enzymes activities (citrate synthase and cytochrome *c* oxidase) and intracellular ATP levels (Figure 3). Therefore, we focused on gallic acid and rutin in the present investigation as potential mitochondrial-activating agents in skeletal muscles. 

Mitochondria are highly dynamic organelles that constantly undergo biogenesis, fusion/fission, and autophagy/mitophagy processes, which determines mitochondrial integrity and functions [44]. SIRT1 is an NAD^+^-dependent histone deacetylase that regulates mitochondrial biogenesis in skeletal muscle. Previous studies have indicated that SIRT1 can deacetylate and activate PGC-1α to enhance mitochondrial-related oxidative phosphorylation and gene expression [45]. 5-aminoimidazole-4 carboxamide ribonucleoside (AICAR) and resveratrol are master regulators of mitochondrial biogenesis (via PGC-1α), which can manipulate mitochondrial metabolism (including oxidative phosphorylation, mitochondrial DNA synthesis, and ATP generation). SIRT1 as an important modulator of muscle metabolism, through its deacetylase activity, regulates the activity of the AMP activated protein kinase (AMPK), transcriptional co-activator, peroxisome proliferator activated receptor-gamma coactivator-1α (PGC-1α), tumor proteinp53, and cyclic AMP response element binding protein (CREB), which is an important factor that links cell energy with increased mitochondrial gene transcription and can drive mitochondrial metabolism to adapt and regulate skeletal muscle growth [46]. In addition, transcription factors, including *NRF1*, *ERRα*, and *TFAM* are key regulators of mitochondrial DNA (mtDNA) transcription and replication [26]. It also reported that these transcription factors could up-regulate mitochondrial biogenesis [25,26]. As shown in Figure 4, C2C12 myotubes treated with gallic acid and rutin could markedly increase the gene expressions of mitochondrial biogenesis, oxidative phosphorylation, myosin heavy chain, fusion/fission, and autophagy/mitophagy (*p* < 0.05). In addition, it has been reported that leucine could induce enhanced mitochondrial mass and oxygen consumption through the upregulation of mitochondrial-related genes, such as, *PGC-1α* and *NRF1*, by activating *SIRT1* in C2C12 myotubes [26]. 

Recent studies have shown that gallic acid and rutin regulated various biological functions through increased SIRT1 gene expression [47,48]. Mitochondrial-related gene expression was reduced when the SIRT1 signaling pathway was disrupted by siRNA. In addition, SIRT1 has been reported to regulate several transcription factors (i.e., PGC-1α, NRF1, and TFAM), thereby promoting mitochondrial biogenesis [25,26], [49]. As shown in Figure 5, we used SIRT1 siRNA to knockdown the expressions of mitochondrial-related genes in C2C12 myotubes. Gallic acid and rutin can be virtually eliminated the expressions of mitochondrial-related genes in C2C12 myotubes inhibited by SIRT1 siRNA. This study indicated that the gallic acid and rutin significantly increased the gene expressions of mitochondrial regulatory is mediated in part by SIRT1. It has been reported that daidzein up-regulated the mitochondrial-related genes in C2C12 myotubes by using an SIRT1-specific siRNA. This study indicates that daidzein regulated transcriptional networks through an SIRT1-associated pathway [50]. De Boer et al. also reported that the regulatory effect of polyphenols on SIRT1 is affected by its stability and metabolism [51]. This study has pointed out that the regulation of SIRT1 is affected by the stability and metabolism of polyphenols. In addition, studies believe that the extrapolation of in vitro SIRT1 stimulation results to *in vivo* physiological effects should be cautious. In follow-up research, we have carried out an animal model that explores the molecular mechanism of SIRT1 pathways from muscle tissue in mice.

## 5. Conclusions

**Figure 6:**
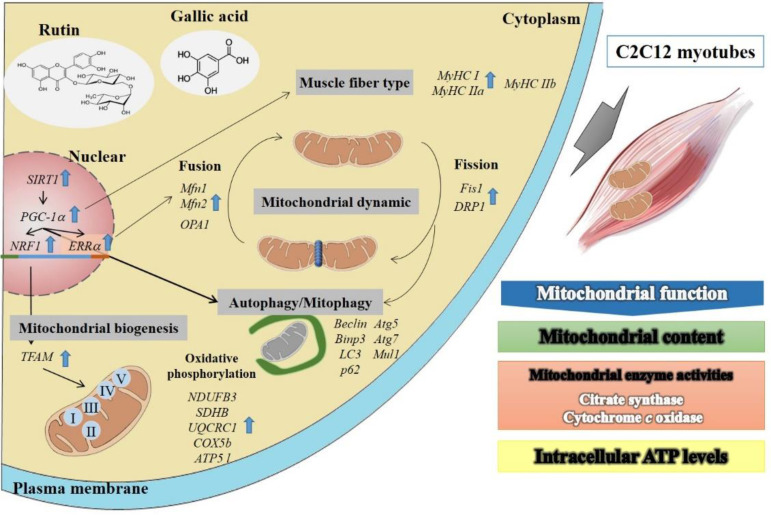
Schematic representation of action mechanism by gallic acid and rutin on mitochondrial functions in C2C12 myotubes. Improvement of functions of the mitochondrial network will result in increased exercise performance. The mitochondria in skeletal muscle are dynamically balanced. By enhancing the performance of genes, such as mitochondrial synthesis, fusion/fission, and mitochondrial autophagy, it can enhance mitochondrial function and improve metabolic dysfunction diseases. Gallic acid and rutin are the most potent activators of mitochondrial function, which is mediated through SIRT1 pathway. The results show that it has the best performance in enhancing the mitochondrial function of C2C12 myotube cells (mitochondrial DNA content, mitochondrial number, enzyme activities, and ATP content). It can further promote the downstream molecular mechanisms of mitochondrial biosynthesis genes (*PGC-1α*, *NRF1*, *ERRα*, and *TFAM*), mitochondrial oxidative phosphorylation (*NDUFB3*, *SDHB*, *UQCRC1*, *COX5b,* and *ATP5 l*), oxidized muscle fibers (*MyHC I* and *MyHC IIa*), fusion/fission (*Mfn1*, *Mfn2*, *OPA1*, *DRP1*, and *Fis1*), and mitochondrial autophagy (*Atg5*, *Atg7*, *Beclin*, *Bnip3*, *LC3II*, *Mul1*, and *p62*) gene expressions to improve skeletal muscle endurance. Therefore, gallic acid and rutin may be beneficial against exercise performance and anti-fatigue action.

Accordingly, this study has demonstrated that gallic acid and rutin increased the gene expression of *SIRT1*, which is crucial for increasing the mitochondrial functions in C2C12 myotubes. In conclusion, our results demonstrated that gallic acid and rutin efficiently increase mitochondrial functions in C2C12 myotubes via up-regulating mitochondrial-related genes of biogenesis, oxidative phosphorylation, myosin heavy chain, fusion/fission, and autophagy/mitophagy (Figure 6). Therefore, gallic acid and rutin may have great potential as novel mitochondrial-activating agents and may play an important role in the development of anti-fatigue functions.

## Data Availability

Not applicable.

## Abbreviations

COX-II, cytochrome c oxidase subunit II; ATP, adenosine triphosphate; SIRT1, silent information regulator two homolog 1; PGC-1α, peroxisome proliferator-activated receptor gamma coactivator-1 alpha; NRF1, nuclear respiratory factor 1; TFAM, mitochondrial transcription factor A; Mfn1, mitofusin 1; LC3II, microtubule-associated protein light chain 3 II; CK, creatine kinase; LDH, lactate dehydrogenase; NAO, 10-N-nonyl acridine orange; ERRα, estrogen-related receptor α.
